# Construction of ultra-high-density genetic linkage map of a sorghum-sudangrass hybrid using whole genome resequencing

**DOI:** 10.1371/journal.pone.0278153

**Published:** 2022-11-29

**Authors:** Qianqian Lu, Xiaoxia Yu, Huiting Wang, Zhuo Yu, Xia Zhang, Yaqi Zhao

**Affiliations:** Agricultural College, Inner Mongolia Agricultural University, Hohhot, Inner Mongolia, China; USDA-ARS Southern Regional Research Center, UNITED STATES

## Abstract

The sorghum-sudangrass hybrid is a vital annual gramineous herbage. Few reports exist on its ultra-high-density genetic map. In this study, we sought to create an ultra-high-density genetic linkage map for this hybrid to strengthen its functional genomics research and genetic breeding. We used 150 sorghum-sudangrass hybrid F_2_ individuals and their parents (scattered ear sorghum and red hull sudangrass) for high-throughput sequencing on the basis of whole genome resequencing. In total, 1,180.66 Gb of data were collected. After identification, filtration for integrity, and partial segregation, over 5,656 single nucleotide polymorphism markers of high quality were detected. An ultra-high-density genetic linkage map was constructed using these data. The markers covered approximately 2,192.84 cM of the map with average marker intervals of 0.39 cM. The length ranged from 115.39 cM to 264.04 cM for the 10 linkage groups. Currently, this represents the first genetic linkage map of this size, number of molecular markers, density, and coverage for sorghum-sudangrass hybrid. The findings of this study provide valuable genome-level information on species evolution and comparative genomics analysis and lay the foundation for further research on quantitative trait loci fine mapping and gene cloning and marker-assisted breeding of important traits in sorghum-sudangrass hybrids.

## Introduction

sorghum-sudangrass hybrid is an important annual gramineous forage hybrid of *Sorghum bicolor* (Linn.) Moench (2*n* = 2*x* = 20) and *S*. *sudanense* (Piper) Stapf (2*n* = 2*x* = 20) [1] with drought tolerance, lodging resistance, and disease resistance characteristics. Moreover, this hybrid has apparent heterosis. Therefore, it has broad application prospects in environmental protection, animal husbandry, and aquaculture, among others. It is widely planted in the United States [2], including northern Colorado [3], China [4], the southern part of the Korean Peninsula, and most of the dry areas of Jeju Island [5]. Dong et al. [6] reported that the sorghum-sudangrass hybrid is rich in amino acids, sugars, minerals, and other compounds, with high nutritional value and good palatability, and is commonly used as hay, silage, or green feed. With the rapid development of animal husbandry, the quality and quantity of existing forage grass fails to meet the growing demand for forage material in developing herbivorous animal husbandry.

An insufficient forage supply has become a limiting factor in the livestock industry in the northwestern Himalayan region of India [7], the southern part of the Korean Peninsula and Jeju Island [5], the northern and southeastern United States [8, 9], and China [10]. Therefore, it is necessary to identify key genes that control desirable traits and select new varieties of the sorghum-sudangrass hybrid with high yields and quality. However, it is challenging to identify genotypes with ideal traits using traditional breeding. One way to overcome this challenge is to construct molecular marker maps on chromosomes using population separation data generated by specific hybridization combinations [11, 12]. Developing a high-density genetic map is necessary for mapping quantitative trait loci (QTLs) in crops and is an essential genetic tool in other areas of molecular biology [13]. At present, domestic and foreign studies on sorghum-sudangrass hybrid mainly focus on cultivation management [14, 15], development and utilization of the nutritional and feeding value [4, 7], or breeding of new varieties [16], However, there are few reports on ultra-high density genetic mapping of the sorghum-sudangrass hybrid.

High-quality molecular genetic linkage maps are prerequisites for the genetic analysis, QTL location of important traits, and efficient molecular breeding (e.g., marker assisted selection [MAS]). The difference types of molecular marker affect the accuracy of genetic linkage maps [17–19]. To date, more than 10 genetic maps of sorghum-sudangrass hybrid were mainly constructed by traditional first- and second-generation types of molecular markers such as amplified fragment length polymorphism (AFLP), restriction fragment length polymorphism (RFLP), simple sequence repeat (SSR), sequence associated amplified polymorphism (SRAP), and randomly amplified polymorphic DNA (RAPD) [20–24]. These maps contained 124–444 molecular markers, spanning 803.13–1468.12 cM, and the average distance between markers ranged from 2.98 cM to 8.84 cM. However, the traditional molecular markers on these genetic maps are based on gel electrophoresis and band counting, which is time-consuming, costly, and prone to human errors. Besides, these maps do not meet the requirements of further fine QTL mapping, as well as annotation and cloning of functional genes, due to the limited number of molecular markers, low map resolution, and large linkage gaps on maps.

In recent years, some high-density genetic linkage maps of plants have been constructed using SNP markers developed by next-generation sequencing (NGS) technology [25]. The rapid development of various genotyping and high-throughput sequencing technologies have led to the development of large-scale SNP markers. In general, the SNP markers used for recently map construction of many plants are mainly based on simplified genome sequencing of NGS technology [26–28]. Jin et al. [29] published a genetic map of sorghum-sudangrass hybrid containing 1,065 markers based on restriction-site-associated DNA sequencing (RAD-seq), which covered a distance of 1,191.7 cM with an average interval of 1.11 cM. Compared with the previously reported maps, the map density here was shortened. However, despite low experimental cost and simple operation of the simplified genome sequencing, some deficiencies still exist, including insufficient sequencing depth, low marker coverage, and the inability to find certain key SNPs loci closely related to the main traits [30, 31].

With the development of NGS and the reduction of sequencing cost, whole genome resequencing (WGRS) technology has become one of the most effective research strategies in breeding and genomics, which has been applied to construct maps of important crops, such as rice [32], tea [31], and potato [33]. However, an ultra-high-density map of sorghum-sudangrass hybrid based on WGRS technology has not been reported.

This study applied WGRS to identify SNPs and build genetic maps of sorghum-sudangrass hybrid, using an F_2_ population obtained by crossing scattered ear sorghum with red hull sudangrass. This ultra-high-density map can be utilized to map QTLs of important traits, perform comparative genomics, and in marker-assisted breeding of sorghum-sudangrass hybrid.

## Materials and methods

The protocol described in this peer-reviewed article is published on protocols.io (http://dx.doi.org/10.17504/protocols.io.14egn2226g5d/v1), and is included for printing as S1 File with this article.

### Plant materials and genomic DNA extraction

**Fig 1:**
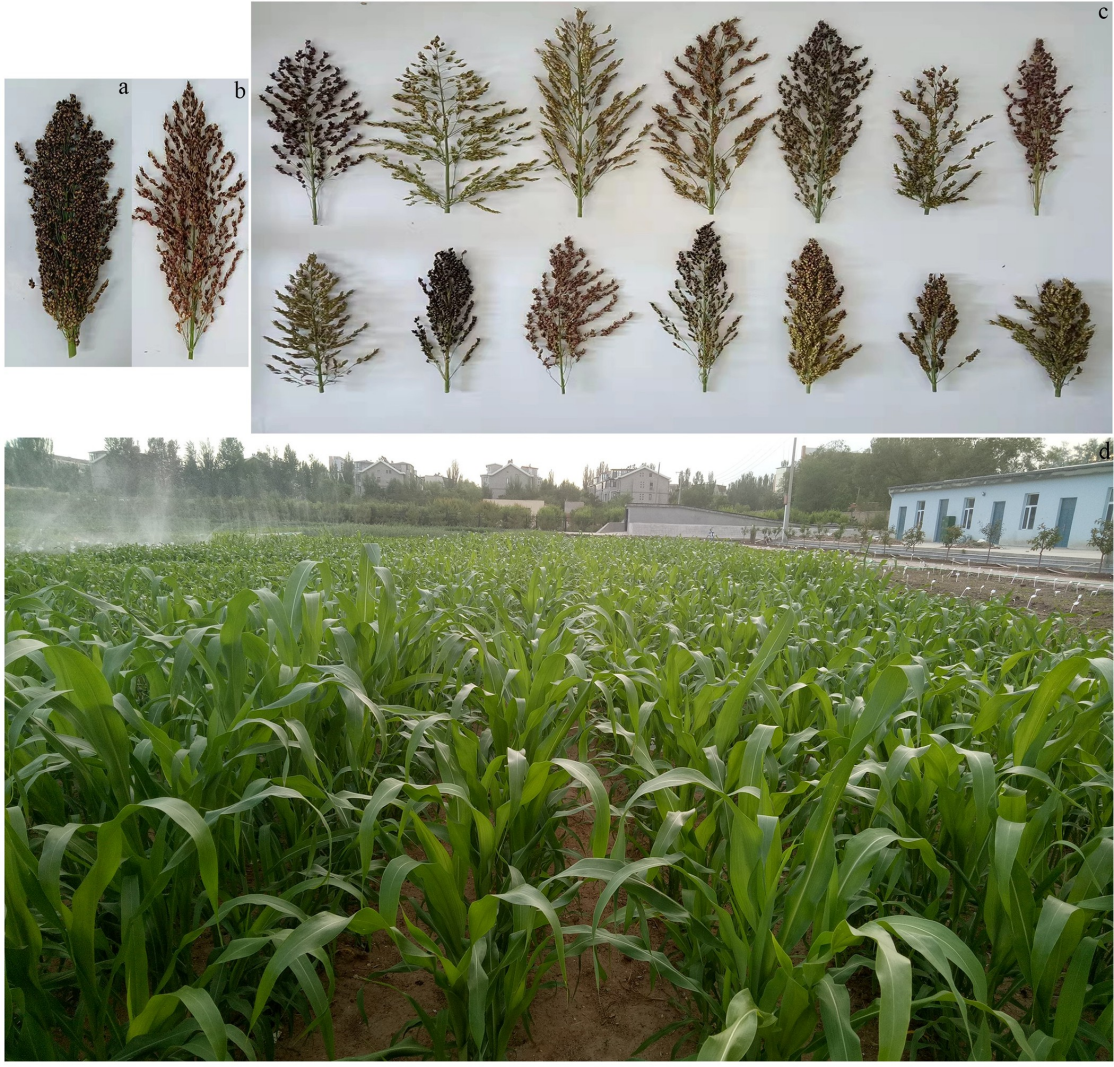
Spike morphology and growth conditions. (a) Comparison of the spike morphology of parent plants. ‘Scattered ear sorghum’ (♀) (left) and ‘red hull sudangrass’ (♂) (right). (b) Spike morphologies of the F_2_ segregating plants of scattered ear sorghum × red hull sudangrass. (c) Growth conditions of the F_2_ segregating plants.

The genetic map was created from a mapping population of 150 individuals selected at random from the F_2_ generation population, which were obtained by self-cross-bagging from F_1_ generation population cross between ‘scattered ear sorghum’ (♀) and ‘red hull sudangrass’ (♂). scattered ear sorghum, a variety with strong drought resistance and high yield, was collected from northeast China. Red hull sudangrass has a high nutritional value and good palatability and is planted widely in Inner Mongolia, China [34]. The experimental materials were planted in the experimental field of the Inner Mongolia Agricultural University, Saihan District, Hohhot (Fig 1). During the early jointing stage, young leaves from the F_2_ individuals and their parents were flash frozen in liquid nitrogen. Genomic DNA (gDNA) was extracted with the DNA secure Plant Kit from Tiangen Biotech, Beijing, China. The quality of the extracted gDNA was confirmed via electrophoresis in a 0.8% (w/v) agarose gel.

### Library construction and genotyping by WGRS

The 152 gDNA samples were randomly sheared into 350 bp fragments in the Covaris breaker (Covaris, Woburn, MA, USA). Libraries were built based on Illumina’s TruSeq Library Construction Kit (Illumina, San Diego, CA, USA). Briefly, the gDNA fragments were processed by end repair, modified by poly-A tail and sequencing adapter addition, purified, and amplified via PCR to construct the library. Paired-end sequencing libraries were sequenced with a read length of 350 bp using an Illumina HiSeqTM PE150 (Illumina, San Diego, CA, USA).

The parental genotypes were sequenced separately at a sequencing depth of 29.71× and 28.77×. Individual F_2_ plants were sequenced at 9.97× coverage. The raw data were filtered to determine the sequencing read quantities, sequencing error rates, Q20, Q30, GC content, and data output. The filtered reads were compared with the reference genome assemblies of sorghum bicolor and used for SNP identification and genotyping.

### SNP calling and genotyping

Sequencing data of the parent and offspring plants were aligned to the reference genome sorghum bicolor (sorghum) (https://phytozome-next.jgi.doe.gov/info/Sbicolor_v3_1_1 Accession ID: ABXC03000000) using Burrows-Wheeler Aligner (BWA) (http://bio-bwa.sourceforge.net/). Duplicate parts (rmDup) were removed by SAMTOOLS (https://www.htslib.org). Marker development was implemented after genotype detection of the parents of sorghum-sudangrass hybrid. Polymorphic markers were obtained after removing the markers of the same type shared by both parents and those missing in one or both parents. GATK (https://gatk.broadinstitute.org) was used to genotype the obtained polymorphic molecular markers, then the markers were then screened out in line with the mapping population, with the conditions of parental 9× and line 3× or 4×, and count the SNPs. In general, polymorphic markers are divided into eight different separation modes, which are comprised of hk × hk, cc × ab, nn × np, aa × bb, lm × ll, ab × cd, ef × eg, and ab × cc. Using the results of parental genotyping, we developed polymorphic markers between the parents, from which we selected those matching the mapping marker type of the population. The percentage of genomic positions covered with at least one and four reads represents 1× and 4× coverage of the reference genome, respectively. sorghum-sudangrass hybrid, as a diploid species, can have a maximum of four alleles per locus. The F_2_ plants were derived from two homozygous parents; hence, the subsequent analysis focused only on SNP markers that showed the aa × bb segregation pattern.

### Genomics map construction with ultra-high-density

SNPs were screened for quality improvement of the genetic map based on the following guidelines: abnormal bases and genotypes were denoted as deletions (indicated by "-"); and Chi-square test was used to identify SNP markers with segregation distortion, which were then filtered out (significance, *p* < 0.001). Linkage maps with 10 linkage groups (LGs) were constructed using Lepmap3 (https://sourceforge.net/projects/lep-map3). After linkage analysis, the obtained high-quality genetic markers, were divided into 10 LGs according to chromosome sequences with LOD values ranged from 2 to 6. The LGs were ordered along the physical location of the chromosomes using the maximum likelihood algorithm, and the genetic distance between markers was calculated using the Kosambi mapping function [35]. Each LG with stable SNPs was aligned to the sorghum_bicolor_v3. reference genome at the cutoff value of 1E^-10^ and sequence coverage rate of >85% using BLASTN (https://blast.ncbi.nlm.nih.gov/) [36]. We evaluated the genetic map of sorghum-sudangrass hybrid using haplotypes and heat maps. Double hybridization and deletion were manifested in the haplotype map as genotyping and labeling sequence errors [37]. Errors in the order of markers were identified using a heat map, reflecting the recombination relationship among the markers of individual LGs. We defined the region with a higher recombination frequency than other regions as the recombination hot spot [38, 39].

## Results

### Statistics and quality assessment of WGRS

A total of 150 F_2_ individuals and their parents were resequenced. There were 1,180.66 GB of sequence data filtered to produce 7,841,459,884 bp of clean reads. (S1 Table) Among all the high-quality data, the number of reads in the males and females was 151,469,838 bp and 156,284,244 bp, respectively. Each individual had 50,224,705.35 reads. The Q20 and Q30 scores for bases between progenies were 97.72% and 93.53%, respectively. The average GC content was 44.37%. The high-quality data and the reference genome comparison showed that the male and female rates were 98.75% and 98.80%. The average rates of the F_2_ generation was 98.58% (Table 1). The results showed that the sequencing data were with high quality and suitable for further analysis.

**Table 1:** Statistics of sequencing data for sorghum-sudangrass hybrid.

	Scattered ear sorghum (♀)	Red hull sudangrass (♂)	F_2_ population		
			Average	Maximum	Minimum
Clean bases (G)	22,720,475,700	23,442,636,600	7,533,705,802	12,622,850,700	6,868,861,500
Clean reads (bp)	151,469,838	156,284,244	50,224,705.3	84,152,338	45,792,410
Mapped reads	149,574,247	154,407,070	49,509,454.1	82,315,401	45,248,627
Mapping rate (%)	98.75	98.80	98.58	99.03	89.55
Q20 (%)	97.06	96.98	97.72	98.06	96.06
Q30 (%)	92.16	91.89	93.52	94.29	90.82
GC content (%)	43.70	43.81	44.37	46.27	43.31
Average depth (×)	28.77	29.71	9.97	8.99	16.07
Coverage 1× (%)	97.05	97.04	94.78	96.08	92.33
Coverage 4× (%)	95.07	95.12	82.56	92.43	77.94
Total SNP	5,365,314	5,199,747	3,191,158.56	4,129,592	2,109,037
Heterozygosis SNP	4,082,017	3,797,357	2,561,679.55	3,361,320	1,450,088
Homozygous SNP	1,283,297	1,402,390	629,479.01	1,173,369	391,753
Het rate (%)	76.08	73.03	80.27	74.12	78.73

The obtained reads were aligned to the sorghum genome, and their genome coverage is shown in Table 1 and S2 Table. Genomic coverage for 1× ranged between 92.33% and 97.05%, with parents covering 1× at 97.05% and 97.04%, and offspring covering 1× at 94.78%. Genome coverage for 4× ranged between 77.94% and 95.12%, with parents having 95.07% and 95.12%, and offspring having 82.56%. The sequencing depth of female scattered ear sorghum and male red hull sudangrass was 28.77× and 29.71× on average, respectively, while the average sequencing depth of the offspring was 9.97×.

Using the GATK tool, we detected 5,199,747 and 5,365,314 SNP markers in scattered ear sorghum and red hull sudangrass, respectively; the number of heterozygous sites was 3,797,357 and 4,082,017, respectively, and the number of homozygous sites was 1,402,390 and 1,283,297, respectively. The heterozygous ratios for scattered ear sorghum and red hull sudangrass were 73.03% and 76.08%, respectively. The average number of SNPs detected in the F_2_ offspring was 3,191,158.56, of which 80.27% were heterozygous (S3 Table). The results showed that the base coverage depth was distributed uniformly across the genome and that the sequencing data were of high quality.

### SNP marker detection and development

A total of 5,633,474 (31.13%) polymorphic markers were obtained (Table 2), which were comprised of hk × hk, cc × ab, nn × np, aa × bb, lm × ll, ab × cd, ef × eg, and ab × cc (Table 3).

**Table 2:** Polymorphism locus statistics.

Marker type	Numbers	Rate (%)
Polymorphic marker	5,633,474	31.13
Missing in parents 1/2/both	4,262,790	23.56
Parents with same type	8,197,958	45.31
Number of input file lines	17,854,886	100

**Table 3:** Segregation distortion for SNP markers.

Segregant type	Cross type	Female genotype	Male genotype	Count	Percentage
Type1	hk × hk	Heterozygosis	Heterozygosis	1,674,553	29.72%
Type2	cc × ab	Homozygous	Heterozygosis	951	0.02%
Type3	nn × np	Homozygosis	Heterozygosis	1,682,365	29.86%
Type4	lm × ll	Heterozygosis	Homozygosis	1,988,538	35.30%
Type5	aa × bb	Homozygosis	Homozygosis	270,715	4.81%
Type6	ab × cc	Heterozygosis	Homozygosis	1137	0.02%
Type7	ab × cd	Heterozygosis	Heterozygosis	8	0.00%
Type8	ef × eg	Heterozygosis	Heterozygosis	15,207	0.27%

Among them, the hk × hk polymorphism site was heterozygous for both parents, the aa × bb site was homozygous for both parents, the hk × hk, ab × cd, and ef × eg sites were heterozygous for both parents, and the nn × np, lm × ll, nn × np, and cc × ab sites were heterozygous for one and homozygous for the other parent. As shown in Table 3, of the 5,633,474 markers, SNPs were most abundant in the lm × ll type (35.30%), followed by that of the nn × np type (29.86%), and only 8 SNPs were in the ab × cd type. Since the mapping population in this study originated from two homozygous parent hybrid plants, the available marker type for the F_2_ population was of the aa × bb type. Subsequently, 270,715 aa × bb progenies were screened for genotype deletion, genotyping error, insufficient completeness (gene coverage ≥75%), and partially separated markers (*p* < 0.001) (S4 and S5 Tables); 8,009 valid SNP markers were obtained after the screening. These were used for linkage analysis of the genetic map of sorghum-sudangrass hybrid.

### Ultra-high-density genetic map construction

**Fig 2:**
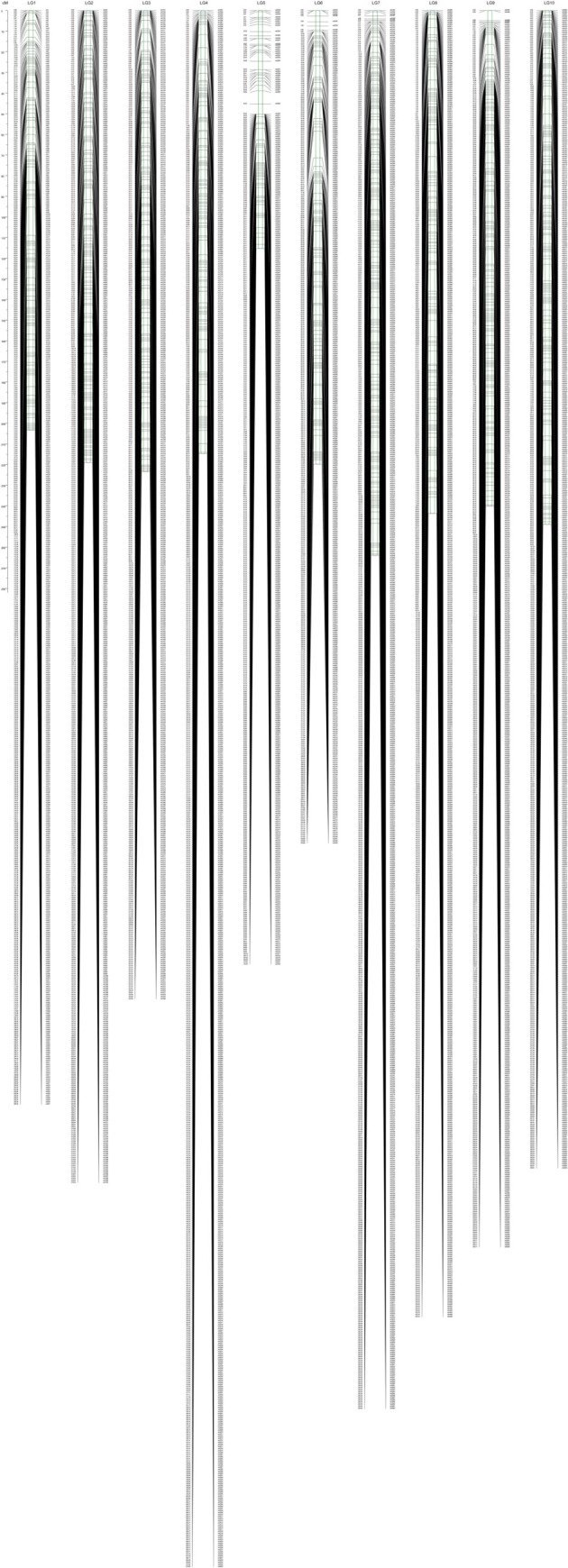
Ultra-high-density genetic linkage map of sorghum-sudangrass hybrid.

Of the 8,009 SNPs identified, subsequent screening of abnormal bases and genotypes, and segregation distortion of unqualified markers produced 5,656 high-quality markers. Using these SNPs, we constructed a high-density genetic map. Each marker was evenly distributed across the 10 LGs (Fig 2). These markers spanned 2,192.84 cM, with an average distance of 0.39 cM between adjacent markers. Approximately 401 to 755 markers were found in each LG, covering a distance of 115.39 cM to 264.04 cM. The number of SNP markers, the total genetic distance, and the average genetic distance on each LG are presented in Table 4 and S6 Table. Among the 10 LGs, the longest was LG7 at 264.04 cM, and the shortest was LG5 at 115.39 cM. The lowest density between markers was observed in LG5, containing 447 SNP markers, with a span of 115.39 cM and the average distance of 0.26 cM between the markers. LG1 had the maximum gap distance between markers of 13.72 cM. In the 10 LGs, 46 intervals had a spacing of 5‒10 cM, three intervals had a spacing of 10‒20 cM, and 5,607 intervals had a spacing of <5 cM. In LG9, up to 99.67% of the intervals between markers was <5 cM. Compared with the map published by Jin et al. [29], our maps are superior in terms of the mapping population size (150 here vs. 126 in [29]), high-throughput sequencing method (WGR-seq vs. RAD-seq), markers number (5,656 vs. 1,065), and sequencing data (7,841,459,884 vs. 233,669,757). Compared to other published genetic linkage maps for sorghum-sudangrass hybrid, our linkage map had the longest total map length (2,192.84 cM) and the shortest mean genetic interval between markers (0.39 cM/ marker) (Table 5). There were three gaps with inter-marker genetic distances greater than 10 cM, among which the largest was 13.72 cM (LG10). Gap distances <5 cM accounted for 99.13% of the total LGs. Overall, the marker distribution of the map was relatively uniform, and the quality of the map was high.

**Table 4:** Characteristics of genetic linkage groups of sorghum-sudangrass hybrid.

Linkage group	Marker number	Length covered	Average length	Max gap	Gap <5 cM	Gap 5–10 cM	Gap 10–20 cM	Gap >20 cM	Ratio
LG1	531	203.34	0.38	13.72	528	2	1	0	99.44
LG2	569	219.04	0.38	6.39	565	4	0	0	99.30
LG3	480	223.58	0.47	7.15	477	3	0	0	99.38
LG4	755	214.69	0.28	9.52	747	8	0	0	98.94
LG5	447	115.39	0.26	6.39	445	2	0	0	99.55
LG6	401	219.86	0.55	12.85	392	8	1	0	97.76
LG7	679	264.04	0.39	9.52	676	3	0	0	99.56
LG8	634	243.72	0.38	10.33	625	8	1	0	98.58
LG9	598	240.17	0.40	7.16	596	2	0	0	99.67
LG10	562	249.01	0.44	7.93	556	6	0	0	98.93
Total	5656	2192.84	0.39	13.72	5607	46	3	0	99.13

**Table 5:** Statistics of the existing map of sorghum-sudangrass hybrid.

Mapping population	Marker types	Markers number	Length (cM)	Average distance (cM)	Linkages	References
F2:3	AFLP, RAPD	166	836	5.03	10	Lu, 2005 [20]
F2	SSR	124	1096.2	8.84	10	Li, 2015 [21]
F2	SSR	181	803.13	4.38	10	Yu, 2018 [22]
F2	AFLP	444	1468.12	3.38	10	Shi, 2017 [23]
F2	SRAP, SSR	427	1273.4	2.98	10	Shi, 2018 [24]
RIL	SNP	1065	1191.7	1.11	10	Jin, 2021 [29]

### Genetic and physical map comparisons and collinearity analyses

Sorghum genome is 3‒4 times smaller than that of maize, and is considered the model plant for polyploid sugarcane and diploid crops [40]. The sorghum-sudangrass hybrid genome was compared to the sorghum genome by Yang et al. [41] and Jin et al. [29], who demonstrated that it is feasible to develop SNP markers for sorghum-sudangrass hybrid using the Sorghum genome. Since the sequencing of the sorghum-sudangrass hybrid genome was incomplete, we used the sorghum genome as a reference genome to develop SNP markers for sorghum-sudangrass hybrid.

**Fig 3:**
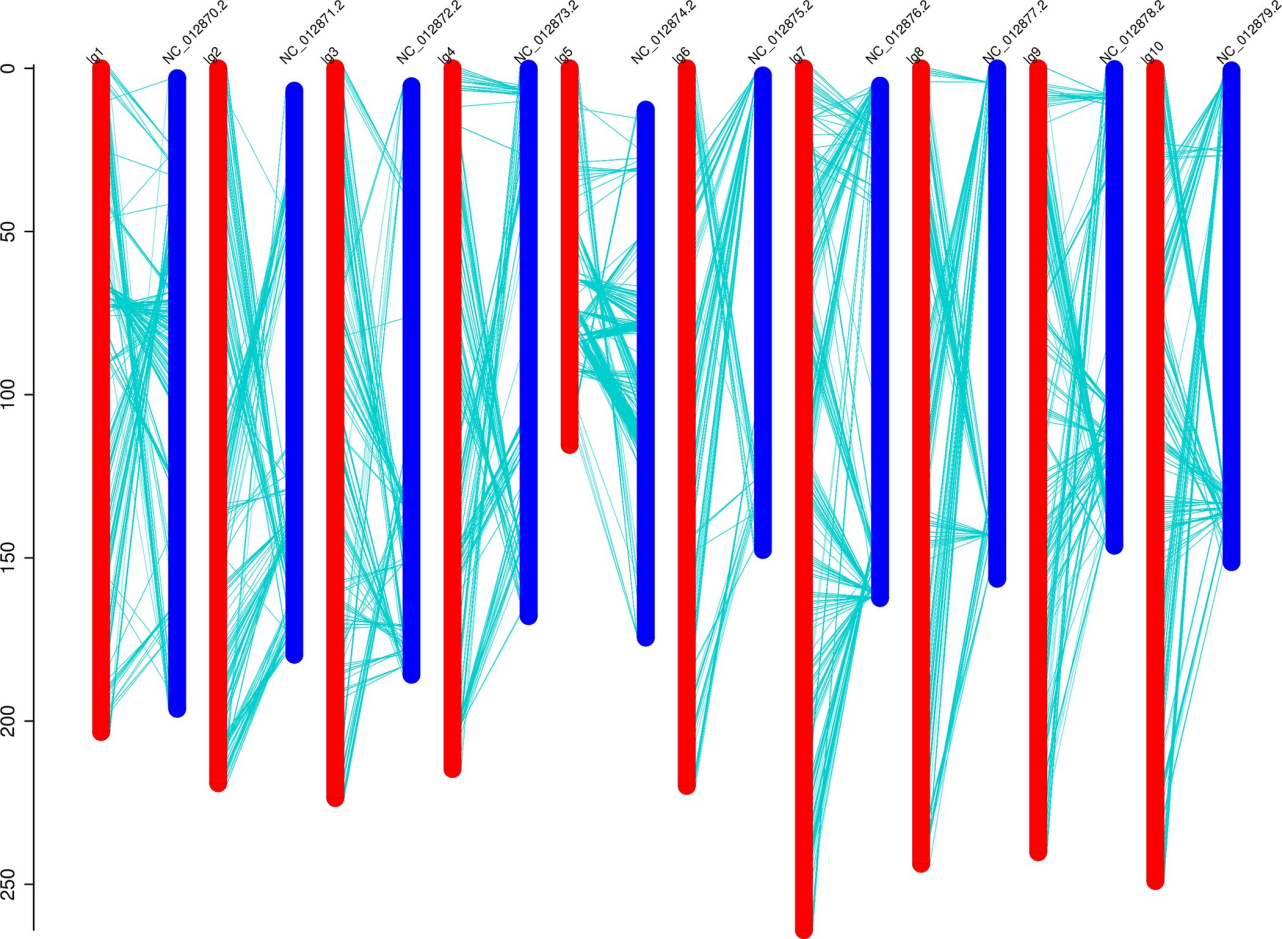
Collinearity analysis between genetic and physical maps of sorghum-sudangrass hybrid. The scale on the left is the relative genetic distance. Red is the genetic map, and blue is the physical map. Blue lines indicate the relationship between map positions.

Quality of a genetic linkage map is judged by the relationship between the SNP marker genetic and physical location [42]. We aligned all 5,656 SNP markers to the reference genome and conducted collinearity analyses. The 10 LGs showed high collinearity, covering almost the entire physical length of the reference genome (Fig 3). The genetic linkage map of most regions matched the reference genome, while short reversals and translocations existed in few areas. Chromosome inversions in individuals that are the product of selfing in a population are important for plant evolution, in particular their adaptation and speciation [37]. This result may guide studies on the utilization of heterosis and identification of hybrids.

### Qualitative analysis of ultra-hight-density genetic maps

In this study, haplotype maps were constructed for 150 F_2_ individuals using 5,656 SNP markers, with almost all the markers clearly defined on the LGs (S1 Fig). The probability of double crossover rate and deletions was extremely low, indicating ithigh map quality. We constructed the heat maps of the 10 chromosomes of sorghum-sudangrass hybrid. The sequence of the 10 LG markers showed the same trend (a red diagonal line in S2 Fig), indicating the high quality of the map.

## Discussion

An increase in the number and type of markers is an effective way of improving the resolution of a map, accelerating the exploration of new genomic information, and mapping of QTLs for important agronomic traits [38, 43]. WGRS represents a new technique to exploit SNPs in plant genomes efficiently. The linkage maps with high densities of various economically important crops have been extensively constructed using WGRS [44–48].

The advantages of WGRS are two-fold. First, compared with marker-based genotyping, maps can be constructed faster and more accurately [34]. Large-scale SNPs developed by WGRS can compensate for the deficiency of marker-based genotyping methods in map construction. Second, WGRS is superior to simplified genome sequencing technologies regarding marker development. Because it uses ultrasonic technology to randomly shear the genomic DNA, it is not restricted by limited restriction enzyme cutting sites. The marker polymorphism is developed with high density, stable inheritance, and easy automation analysis. With the increasing advancement in DNA sequencing platforms, WGRS technology has become the primary choice of NGS.

Recently, the genomes of sesame [44], *Brassica napus* [45], peanut [46], and soybean [47] were mapped using WGRS. Lin et al. [48] implemented WGRS to develop a high-density genetic map of wheat and identified 21,197 polymorphic SNP markers. In the present study used WGRS to sequence 150 F_2_ plants and their parents. The sequencing data were compared with the reference genome, and many SNP markers were detected, greatly improving the map’s quality. After rigorous screening and filtering, 5,656 high-quality polymorphic SNPs were obtained, and a high-density genetic linkage map of sorghum-sudangrass hybrid was constructed. Compared to other published genetic linkage maps for sorghum-sudangrass hybrid, the one presented herein contains the most SNP markers and the highest marker density. Therefore, WGRS is an efficient and accurate strategy for marker development and construction of genetic maps on a large scale.

Several genetic maps were created for sorghum-sudangrass hybrid (Table 5), but important quantitative traits could not be accurately identified due to the limitations pertaining to the available molecular markers and low resolution of the maps. The earliest map comprised 158 AFLP and 8 RAPD markers, covering 836 cM of the sorghum-sudangrass hybrid genome and with the average marker spacing of 5.03 cM [20]. In the present study, we constructed an ultra-high-genetic linkage map containing 5,656 SNP markers. Compared with previous maps, our maps are superior in terms of the mapping population size, high-throughput sequencing method, markers number, and sequencing data [20–24, 29]. The genome coverage of our map was greatly improved, which may be attributed to several reasons: (1) The large genetic diversity between the two parents used in this study contributed to their significant difference in several important traits (Fig 1). The research showed that parents with extreme differences in one or more phenotypic traits are ideal for mapping populations because it lays a foundation for QTL mapping of important traits and construction of high-density genetic maps [49]. (2) The sequencing depth was deep enough. To obtain sufficient SNP markers, we sequenced the parents and progeny at a depth of 28.77×, 29.71×, and 9.97×. (3) The WGRS approach allowed for a development of a large number of SNP markers in the genome. It increases map density, makes further QTL detection more accurate and accelerates the screening process of candidate genes related to important traits in plants. In addition, this map can provide high-quality references for future research on molecular genetics and genomics of sorghum-sudangrass hybrid.

High-resolution linkage mapping has become an indispensable part of genetic research. It can effectively identify important agronomic QTLs and plays important roles in fine mapping, candidate gene prediction, and marker-assisted breeding [13].

The high-density genetic map is a valuable tool to study plant phenotypic variation and shorten the breeding cycle. It is a bridge between traits and genomes, revealing the relationship between genes and phenotypic variation [50]. In recent years, with the increasing number of available complete genome sequences of crops, high-density genetic maps have been combined with crop genomes to identify QTL loci physically similar to the genome [51]. The high-density genetic maps have been widely used in QTL mapping, fine mapping of important traits, and candidate gene prediction in weed-rice [51], wheat [52], maize [53], rape [54], cotton [55], and others. In addition, high-density genetic maps can also be served as a platform for genome assembly or studing the collinearity between related species and the structural variation of hybrid genomes [40].

The ultra-high-density genetic map constructed in this study has the largest number and highest density of markers that is currently available for the sorghum-sudangrass hybrid. The population sample size directly affects the aquality and accuracy of genetic map. However, due to human and material resource limitations, selecting an appropriate population size is critical, with most studies using 100‒200 progeny or individuals [56]. Our study developed an ultra-high-density genetic map with 5,656 SNP markers from 150 plants of an F_2_ sorghum-sudangrass hybrid population. Next, we will apply these markers to a larger population to increase the map resolution. This will lay a foundation for fine mapping of QTLs for important traits in sorghum-sudangrass hybrid.

## Conclusions

High-density genomic maps can optimize marker-assisted breeding in sorghum-sudangrass hybrid, which utilizes molecular markers linked to or separated from target traits. sorghum-sudangrass hybrid is an important annual gramineous forage that requires further improvement. Marker-assisted breeding is more effective when the genetic map is saturated. We have constructed an ultra-high-density linkage map for sorghum-sudangrass hybrid for the first time, utilizing the SNP markers developed using the WGRS. A map containing 5,656 SNP markers was constructed, covering an area of 2,192.84 cM with an average spacing between markers of 0.39 cM. sorghum-sudangrass hybrid breeding will be greatly improved with this ultra-high-density genetic map, which offers an improvement upon existing maps. Furthermore, this study is of great importance for developing diploid sorghum-sudangrass hybrids. The application of WGRS will provide a valuable reference for other diploid graminoid crops.

## Supporting information

S1 Fig Haplotype maps for 10 linkage groups of the sorghum-sudangrass hybrid. From chromosomes 1 to 10, ten haplotype maps represent an individual’s genotype. Each row of numbers on the left represents a sample number. The horizontal axis represents the tag name. There are two chromatids from each parent in green and blue; missing data are shown in red and heterozygosity in purple.
(TIF)

S2 Fig Heat maps for 10 linkage groups of the density genetic map for the sorghum-sudangrass hybrid. Ten heat maps are shown from chromosome 1 to chromosome 10, in which markers are listed alphabetically by row and column. Different colors indicate the strength of linkage: yellow represents weak links, whereas red represents strong links.
(TIF)

S1 Table Statistics of population resequencing in sorghum-sudangrass hybrid. (XLS)

S2 Table Population resequencing and coverage statistics. (XLS)

S3 Table Statistics of parental SNP detection results. (XLS)

S4 Table Statistics of parents and F_2_ population aa × bb marker with 9× 3×. (XLS)

S5 Table Statistics of parents and F_2_ population aa × bb marker with 9× 4×. (XLS)

S6 Table High density genetic map of sorghum-sudangrass hybrid. (XLS)

S1 File Construction of ultra-high-density genetic linkage map of a sorghum-sudangrass hybrid. Also available on protocols.io. http://dx.doi.org/10.17504/protocols.io.14egn2226g5d/v1.
(DOC)
