# Supplementary figures and images for: Construction of ultra-high-density genetic linkage map of a sorghum-sudangrass hybrid using whole genome resequencing
Source: PLoS One. 2022 Nov 29;17(11):e0278153. doi: 10.1371/journal.pone.0278153 (PMC9707794; doi:10.1371/journal.pone.0278153)

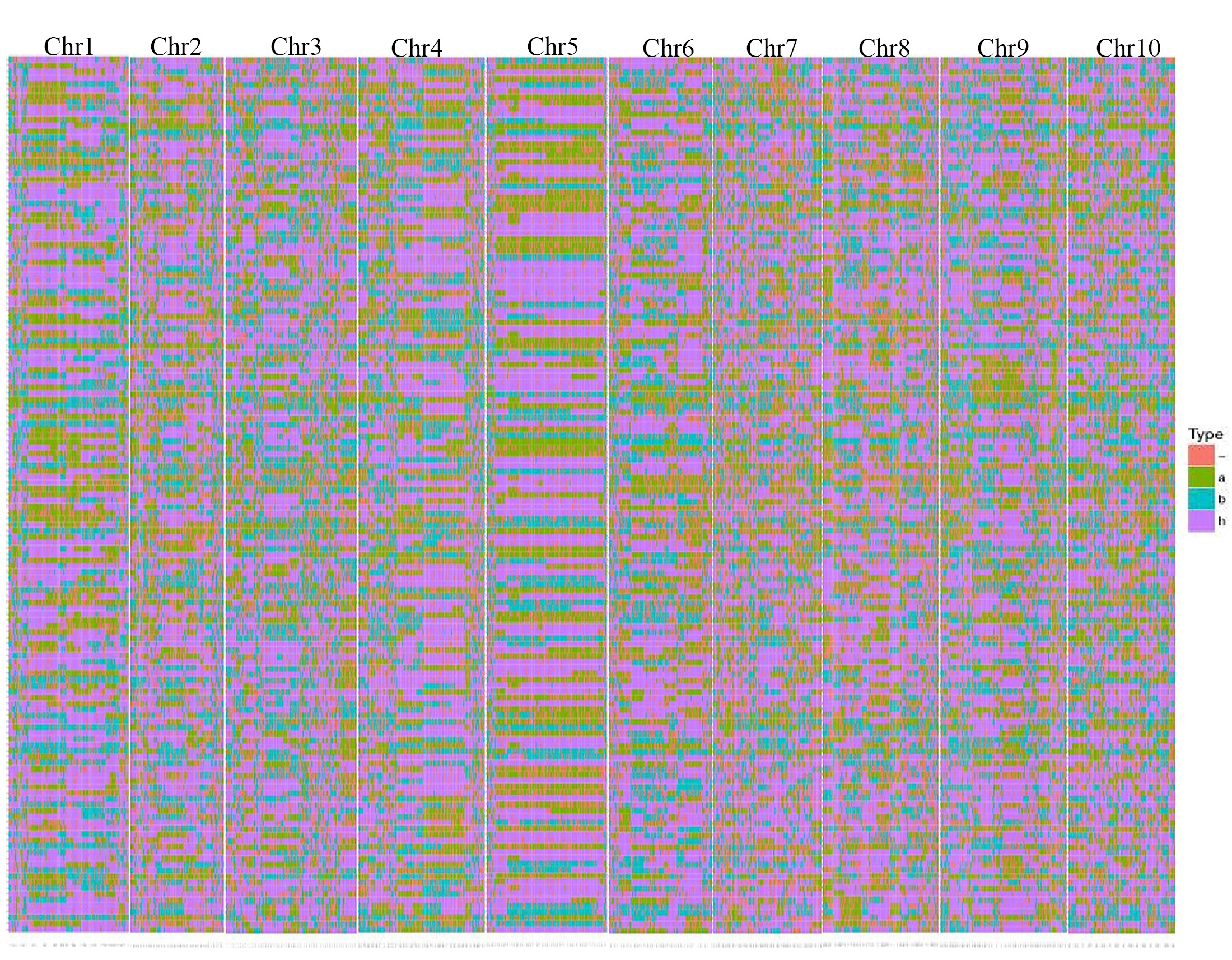

Supplement: S1 Fig — From chromosomes 1 to 10, ten haplotype maps represent an individual’s genotype. Each row of numbers on the left represents a sample number. The horizontal axis represents the tag name. There are two chromatids from each parent in green and blue; missing data are shown in red and heterozygosity in purple. (TIF) [file pone.0278153.s001.tif]

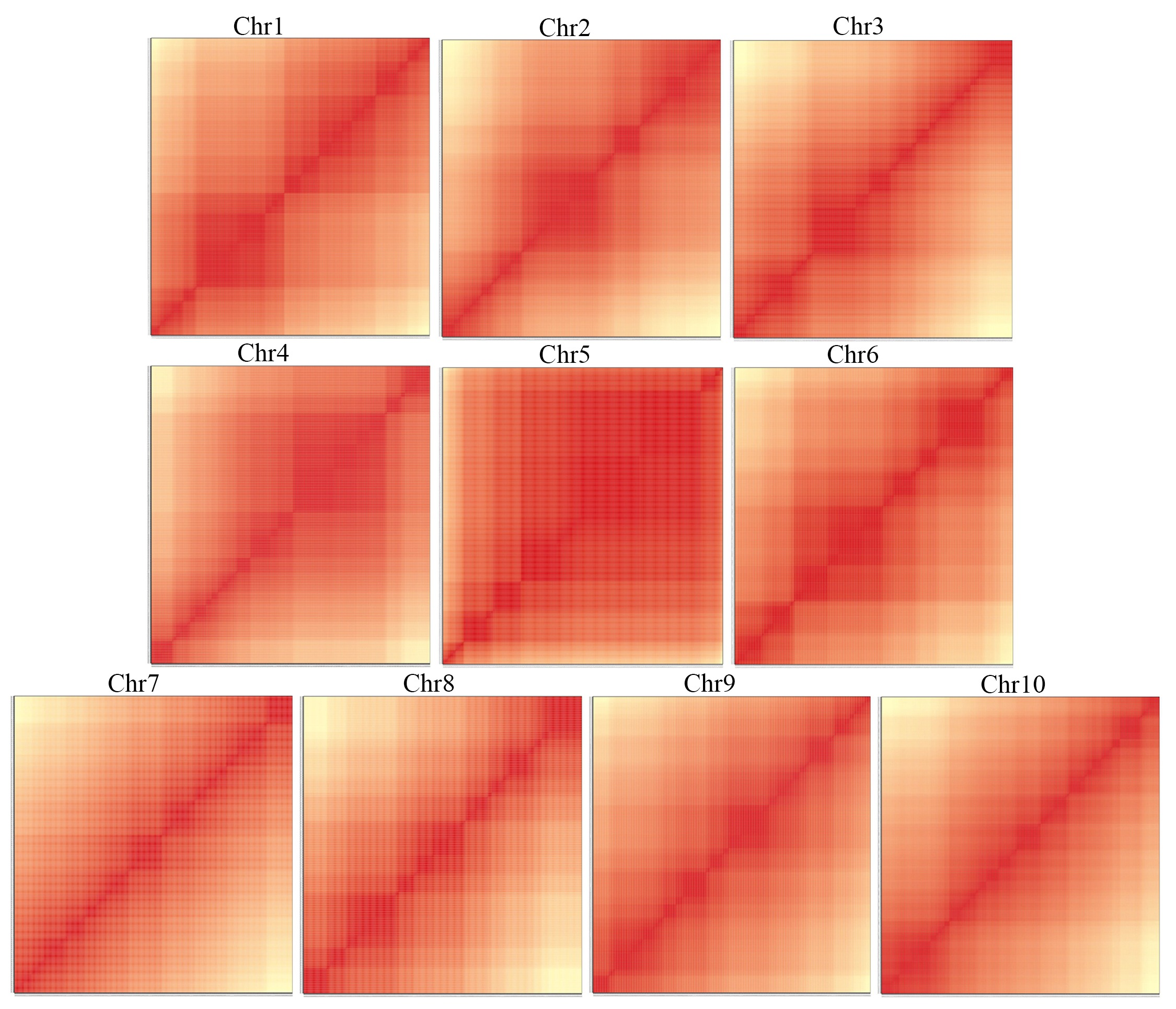

Supplement: S2 Fig — Ten heat maps are shown from chromosome 1 to chromosome 10, in which markers are listed alphabetically by row and column. Different colors indicate the strength of linkage: yellow represents weak links, whereas red represents strong links. (TIF) [file pone.0278153.s002.tif]
