# Supplementary material for: Construction of ultra-high-density genetic linkage map of a sorghum-sudangrass hybrid using whole genome resequencing
Source: PLoS One. 2022 Nov 29;17(11):e0278153. doi: 10.1371/journal.pone.0278153 (PMC9707794; doi:10.1371/journal.pone.0278153)
Supplement: S1 File — Also available on protocols.io. http://dx.doi.org/10.17504/protocols.io.14egn2226g5d/v1. (DOC) [file pone.0278153.s009.doc]

Construction of ultra-high-density genetic linkage map of a sorghum-sudangrass hybrid using whole genome resequencing

Abstract

The sorghum-sudangrass hybrid is a vital annual gramineous herbage. Few reports exist on its ultra-high-density genetic map. In this study, an ultra-high-density genetic linkage map was constructed using these data. Currently, this represents the first genetic linkage map of this size, number of molecular markers, density, and coverage for Sorghum-sudangrass. The findings of this study provide valuable genome-level information on species evolution and comparative genomics analysis and lay the foundation for further research on quantitative trait loci fine mapping and gene cloning and marker-assisted breeding of important traits in Sorghum-sudangrass hybrids.

Materials and methods

Plant materials

The genetic map was created from a mapping population of 150 individuals selected at random from the F_2_ generation population, which were obtained by self-cross-bagging from F_1_ generation population cross between ‘scattered ear sorghum’(♀) and ‘red hull sudangrass’(♂).

Genomic DNA extraction

During the early jointing stage, young leaves from the F_2_ individuals and their parents were flash frozen in liquid nitrogen. The quality of the extracted gDNA was confirmed via electrophoresis in a 0.8% (w/v) agarose gel.

Library construction and genotyping by WGRS

The 152 gDNA samples were randomly sheared into 350 bp fragments in the Covaris breaker (Covaris, Woburn, MA, USA). Libraries were built based on Illumina's TruSeq Library Construction Kit (San Diego, CA, USA). Briefly, the gDNA fragments were processed by end repair, modified by poly-A tail and sequencing adapter addition, purified, and amplified via PCR to construct the library. Paired-end sequencing libraries were sequenced with a read length of 350 bp using an Illumina HiSeqTM PE150 platform.

SNP calling and genotyping

Sequencing data of the parent and offspring plants were aligned to the reference genome sorghum bicolor (sorghum) (https://phytozome-next.jgi.doe.gov/info/Sbicolor_ v3_1_1 Accession ID: ABXC03000000) using Burrows-Wheeler Aligner (BWA) (<http://bio-bwa.sourceforge.net/>). Duplicate parts (rmDup) were removed by SAMTOOLS (https://www.htslib.org). GATK (https://gatk.broadinstitute.org) was used to genotype the obtained polymorphic molecular markers, the markers were then screened out in line with the mapping population, with the conditions of parental 9× and line 3× or 4×, and count the SNPs.

Genomics map construction with ultra-high-density

SNPs were screened for quality improvement of the genetic map based on the following guidelines:

1. abnormal bases and genotypes were denoted as deletions (indicated by "-"); and Chi-square test was used to identify SNP markers with segregation distortion, which were then filtered out (significance, p < 0.001).

(2) Linkage maps with 10 linkage groups (LGs) were constructed using Lepmap3 (https://sourceforge.net/projects/lep-map3). After linkage analysis, the obtained high-quality genetic markers, were divided into 10 LGs according to chromosome sequences with LOD values ranging from 2 to 6.

(3) The LGs were ordered along the physical location of the chromosomes using the maximum likelihood algorithm, and the genetic distance between markers was calculated using the Kosambi mapping function.

(4) Each LG with stable SNPs was aligned to the sorghum_bicolor_v3. reference genome at the cutoff value of 1E-10 and sequence coverage rate of >85% using BLASTN (https://blast.ncbi.nlm.nih.gov/).

(5) We evaluated the genetic map of sorghum-sudangrass hybrid using haplotypes and heat maps. Double hybridization and deletion were manifested in the haplotype map as genotyping and labeling sequence errors. Errors in the order of markers were identified using a heat map, reflecting the recombination relationship among the markers of individual LGs. We defined the region with a higher recombination frequency than other regions as the recombination hot spot.
